# Tick Diversity and Distribution of Pathogen in Ticks Collected from Wild Animals and Vegetation in Africa

**DOI:** 10.3390/pathogens14020116

**Published:** 2025-01-25

**Authors:** Roland Eric Yessinou, Aldric Koumassou, Haruna Baba Galadima, Hospice Nanoukon-Ahigan, Souaïbou Farougou, Martin Pfeffer

**Affiliations:** 1 Communicable Diseases Research Unit, Department of Production and Animal Health, University of Abomey-Calavi, P.O. Box 01, Cotonou 2009, Benin; koumassoualdric@gmail.com (A.K.); sperosnanoukon@gmail.com (H.N.-A.); souaibou.farougou@uac.bj (S.F.); 2 Department of Veterinary Medicine, University of Maiduguri, Maiduguri 600104, Nigeria; hbgaladima@unimaid.edu.ng; 3 Institute of Animal Hygiene and Veterinary Public Health, Faculty of Veterinary Medicine, University of Leipzig, An den Tierkliniken 1, 04103 Leipzig, Germany; pfeffer@vetmed.uni-leipzig.de

**Keywords:** ticks, bacteria, parasite, virus, wildlife, vegetation

## Abstract

Ticks are important vectors of a wide range of pathogens with significant medical and veterinary importance. Different tick species occupy different habitats with an overall widespread geographical distribution. In addition to their role as reservoirs or vectors, ticks are involved in maintaining pathogens in the environment and among wild and domestic animals. In this study, tick species infesting wild animals, as well as collected from the environment and their pathogens reported in 17 countries in Africa between 2003 and 2023, were collected according to the PRISMA guidelines. Data on ticks resulted in a total of 40 different tick species from 35 different wild animal species. Among the ticks, 34 infectious agents were noted including parasitic (*Babesia*, *Theileria*, *Hepatozoon*, *Eimeria*), bacterial (*Anaplasma*, *Bartonella*, *Borrelia*, *Candidatus* Midichloria mitochondrii, *Candidatus* Allocryptoplasma spp., *Coxiella*, *Ehrlichia*, *Francisella*, and *Rickettsia*), and a surprisingly high diversity of viral pathogens (Bunyamwera virus, Crimean-Congo Haemorhagic Fever virus, Ndumu virus, Semliki Forest virus, Thogoto virus, West Nile virus). These results highlight the public health and veterinary importance of the information on tick-borne infections. This knowledge is essential to strive to implement programs for sustainable control of ticks and tick-borne diseases.

## 1. Introduction

Ticks are hematophagous ectoparasites that infest animals and humans and are able to transmit a wide variety of pathogenic microorganisms (parasites, bacteria, and viruses) [1]. Tick-borne diseases are of great medical and veterinary significance [2]. Although it is evident that the incidence of tick-borne diseases continues to increase as well as their geographical ranges, few studies were conducted on wildlife-infesting ticks and their pathogens [3,4]. Tick-borne infectious diseases constitute a major threat to public health [5], largely due to animal movements and the impact of climate change on ticks and their hosts [6,7,8]. Ticks have a large capacity for adaptation to rural and urban ecosystems thus increasing the risk of infestation of vertebrates living in sympatry with humans [9,10,11]. In addition to forests and national parks, encounters between humans and wild animals (elk, birds, pumas, rats, birds, shrews, lizards, coyotes, black bears, geese, raccoons, and foxes) are increasingly common in urban and peri-urban environments [12,13]. Wild animals can serve as hosts for ticks and also as reservoirs of tick-borne pathogens [14]. Feeding of wild animals by humans (including waste from landfills and artificial watering) has facilitated the successful reproduction of certain wild animal species, which have adapted very well to their urban and peri-urban environments thereby enhancing their invasive behavior [12,15]. The frequency of wild–domestic animal contact could favor the exchange of ticks, in particular species of ticks which have several hosts [16], which in turn would enhance humans’ exposure more, because they share the habitats with their domestic animals [17]. But also, the demographics pushing urbanization and the rapid development of cities have prompted humans to occupy the habitat of wildlife and to live in close contact with these animals, so this cohabitation is not without consequences on human health [18]. Despite the rapid decline in the population of wild animals and the large-scale destruction of their habitat, wildlife plays an important role in the maintenance of ticks as hosts but also in the transmission of parasitic zoonoses in rural and urban areas [18,19,20]. Interactions between wild domestic and human animals increase the risk of tick infestation and exposure to associated pathogens [21,22].

Several emerging tick-borne pathogens can represent a significant threat to human and animal health [23,24,25], and ticks could play an important role in the dynamic and epidemiology of infectious pathogens [26]. Despite the overall increase in the abundance of ticks due to the intensification of agricultural activities, livestock breeding, and domestication of wild animals, the impact of pathogens in wildlife-infested ticks is poorly understood and limited to well-characterized pathogens like *Ehrlichia* or *Babesia* species [27,28]. Therefore, understanding the role of wildlife-infesting ticks in the epidemiology and dynamics of pathogens is crucial due to the potential for the transmission and also the emergence of zoonotic diseases in these last centuries [29].

In addition to migratory birds, which play a role in the dissemination of ticks and their pathogens [30,31], the trade in wild animals also has an important role in the expansion of their geographical range. Tick-borne pathogens were reported in ticks on snakes and lizards of African origin in international trade (Europe) [32]. Wild animals could play an important role in the distribution and establishment of ticks and their pathogens across continents [33]. Several tick-borne pathogens have also been reported in ticks collected from the vegetation; this could be a potential source of disease for walkers and animals passing [33,34,35,36]. Therefore, it is necessary to understand the ecology of ticks infesting wildlife and identify their pathogens in order to judge about the risks of exposure and help to reduce the impact of tick-borne diseases.

## 2. Materials and Methods

### 2.1. Search Strategy

The PRISMA (Preferred Reporting Items for Systematic Reviews and Meta-Analyses) guideline was used for the systematic search of literatures [37]. We searched exhaustively several electronic databases in English and French including PubMed, Science Direct, Cab Direct, Scopus, Web of Science, and Google Scholar databases between 2003 and 2023. Reference Manager^®^ was initially used for title and abstract screening of the articles. The keywords used in online databases to select the articles were as follows: “ticks”, “wild animals”, “wildlife”, “tick borne diseases”, “tick-borne diseases”, “tick borne pathogens”, “tick-borne pathogens”, “tick-borne infectious diseases”, “tick-borne zoonotic pathogens” “PCR”, “RT-PCR”, “Sequencing”, and “Sequence” were used. These key terms were employed either alone or in combination, utilizing Boolean operators such as “AND”, “OR”, and “NOT”. We included publications reporting on tick-borne pathogens from ticks collected in wild animals in Africa. Peer-reviewed, original research in English or French language was included.

### 2.2. Eligibility Criteria

Several criteria were used to select eligible publications: (1) this study was performed on ticks collected from wild animals or the environment; (2) tick-borne pathogens have been identified only by PCR assay. We excluded articles describing tick-borne pathogens on ticks collected from domestic animals, reports on tick-borne pathogens linked to domestic animal hosts (e.g., ruminant, dog, and horse), and if the study was a review, an experimental laboratory study, or had only incomplete data in terms of tick or host taxa or pathogen identity.

### 2.3. Study Characteristics and Data Extraction

The extracted data included year of publication, host, and country of the study, study area, pathogenic agents, and tick species. All titles and abstracts were examined by two authors and full-text articles were retrieved. All data were extracted and subsequently transferred to Excel (Microsoft Corporation, Redmond, WA, USA).

### 2.4. Data Cleaning and Processing

All the literature data regarding tick collection areas, along with their geographic coordinates, were recorded in a database. The georeferenced data were mapped to a decimal degree coordinate system using Google Earth. Analysis of the distribution of ticks and tick-borne pathogens in the study area was performed and digital maps were created using QGIS version 3.10.

## 3. Results

### 3.1. Outcome of the Literature Search

**Figure 1:**
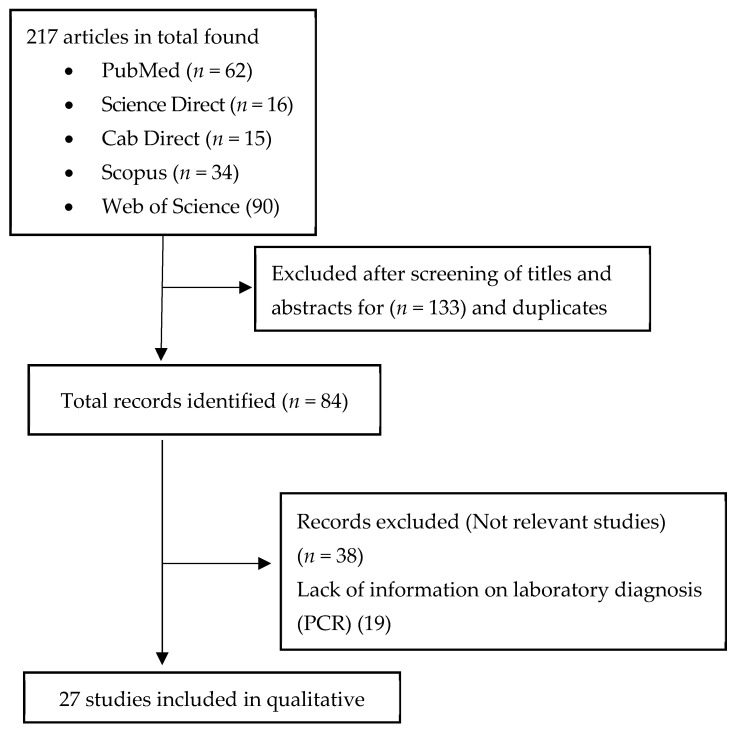
PRISMA flow diagram describing the process of selecting eligible studies for review systematic on pathogens identified in ticks collected in wildlife and vegetation.

A total of 217 articles were identified in the initial searches in Science Direct, PubMed, Cab Direct, Scopus, Web of Science, and Google Scholar databases. Titles and abstracts of retrieved publications were evaluated regarding the inclusion and exclusion criteria. After removing duplicates, 182 titles and abstracts were screened, of which 98 were excluded. The full texts of the remaining 84 records were assessed and 57 articles were deleted. After the final screening, 27 research articles were included in this review (see Figure 1).

### 3.2. Characteristics of the Eligible Studies Included in the Systematic Review

Details on the characteristics of included studies are provided in Table 1. The literature survey of pathogens and their known (suspected) vectors and reservoir hosts were reported in 17 countries of Africa. Of the 27 studies, 13 were reported in North Africa, mainly in Algeria, Morocco, and Tunisia; however, in Sub-Saharan Africa, 14 studies were documented in some countries such as Benin, Ethiopia, Gambia, Ghana, Kenya, Liberia, Mali, Mauritania, Mozambique, Senegal, South Africa, Tanzania, Uganda and Zambia. Overall, 40 species of ticks were collected from 34 wild animal species. Ticks mainly belong to the genera *Amblyomma* (14), followed by *Rhipicephalus* spp. (9), *Hyalomma* spp. (6), *Ixodes* spp. (4), *Ornithodoros* spp. (3), *Haemaphysalis* spp. (3), and a single *Dermatocentor* species.

A total 34 infectious agents were detected on the wildlife-infesting ticks including parasites (*Babesia* spp., *Theileria* spp., *Hepatozoon* spp.), bacteria (members of the genera *Anaplasma*, *Bartonella*, *Borrelia*, *Coxiella*, *Ehrlichia*, and *Rickettsia*), and a surprisingly high variety of viruses (Bunyamwera virus, Crimean-Congo Haemorrhagic Fever virus, Ndumu virus, Semliki Forest virus, Thogoto virus, West Nile virus; see Table 1).

Twelve tick species were reported after collection from vegetation belonging to the five genera *Amblyomma*, *Haemaphysalis*, *Hyalomma*, *Ixodes*, and *Rhipicephalus*. Pathogens identified were mainly *Anaplasma*, *Babesia*, *Borrelia*, *Candidatus* spp., *Ehrlichia*, *Rickettsia*, and *Theileria* (Table 2).

**Figure 2:**
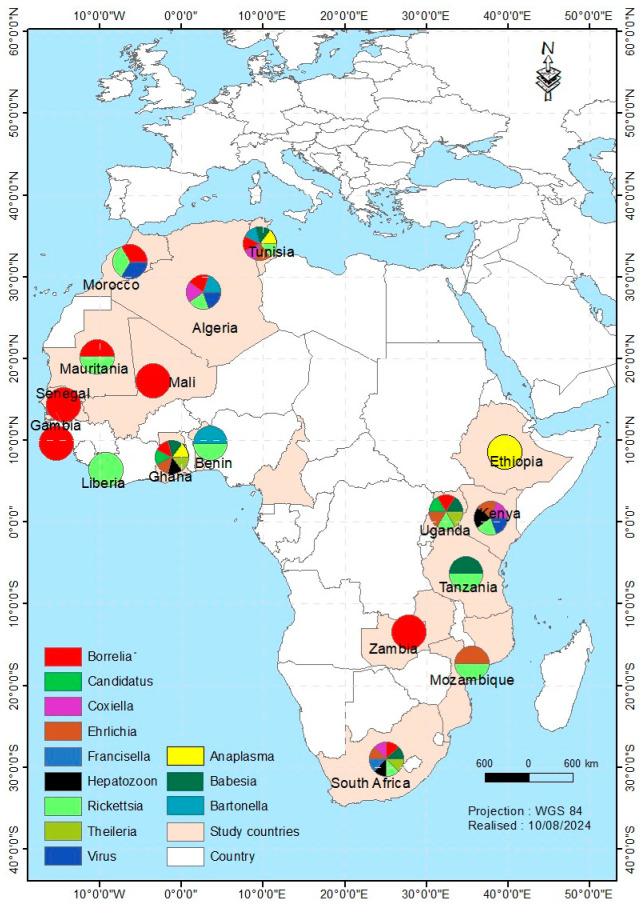
Pathogens reported in wildlife-infesting ticks in Africa. Candidatus is either Midichloria mitochondrii or *Allocryptoplasma* spp. Virus is either CCHFV, Ndumu-, Bunyamwea-, Semliki Forest-, West Nile-, or Thogoto virus.

Pathogen infection can be transstadial and transovarial or acquired during the blood meal of tick from the host. Several tick-borne pathogens were reported in the studies reviewed. Among the reported pathogens, two species of *Anaplasma* were detected, *Anaplasma bovis* reported in *Hyalomma dromedarii* while *Anaplasma phagocytophilum* was reported from *Hyalomma aegyptium* and *Ixodes aulacodi*. *Bartonella tamiae* infection was associated with *Ixodes vespertilionis* while *Ornithodoros erraticus* and *Ixodes ricinus* ticks were associated with *Borrelia crocidurae* and *Borrelia lusitanae*, respectively. Soft ticks harbored *Rickettsia* species, including *Rickettsia bellii*, *Rickettsia felis*, *Rickettsia hoogstraalii*, *Rickettsia nicoyana*, *Rickettsia wissemanii*, and *Rickettsia asemboensis*, which were found in *Ornithodoros occidentalis*, *O. erraticus*, and *Ornithodoros normandi* collected from wild animals. Furthermore, *Rickettsia africae* was identified in *Amblyomma lepidum*, *Amblyomma compressum*, and *Amblyomma variegatum* while *Rickettsia aeschlimannii* was reported in *H. aegyptium* and *Hyalomma impeltatum. Coxiella burnetii* is widespread and reported in *I. vespertilionis*, *Haemaphysalis erinacei*, *Rhipicephalus sanguineus*, and *H. aegyptium*. Results showed that *Ehrlichia ruminantium* infected *A. sparsum*, *Amblyomma eburneum*, *A. variegatum*, *Amblyomma falsomarmoreum*, and *Amblyomma nuttalli*. Several viruses were detected in *Rhipicephalus pulchellus* including Bunyamwera virus, Ndumu virus, Thogoto virus, and Semliki Forest virus, but Crimean-Congo Haemorrhagic Fever virus was found only in *H. aegyptium* and *Hyalomma marginatum*. Several species of wild-living mammals and birds are exposed to infestation of ticks in nature. Here, tick infestations reported in Addax antelope mainly concerned species of *A. variegatum*, *R. pulchellus*, and *H. dromedarii*. Ticks recorded in rodent burrows included *O. occidentalis*, *O. erraticus*, and *O. normandi*. *R. sanguineus* was shown to be a tick that has infested a wide variety of wild animals. It was reported from African wildcat, Gambian pouched rat, grasscutters, hedgehogs, mongooses, Rüppell’s fox, and wild boars in several countries. Among *Amblyomma* species reported, *Amblyomma sylvaticum*, *Amblyomma sparsum*, *Amblyomma marmoreum*, *H. aegyptium*, *A. nuttalli*, and *A. falsomarmoreum* were identified in association with tortoises while *Amblyomma latum* was observed in Ball python, not further specified snakes from South Africa, and Monitor lizards. In addition to *A. marmoreum*, *Amblyomma exornatum* found in Monitor lizards, *Amblyomma transverse*, *Amblyomma gemma*, *A. compressum*, and *A. lepidum* were identified in Ball python, giraffe, warthog, pangolin, and white rhinoceros. Species of *I. vespertilionis*, *Ixodes rasus*, *Ixodes muniensis*, *I. aulacodi*, and *I. ricinus* were reported in bats, chimpanzee, duiker, grasscutters, and Monitor lizards, respectively. The results showed that *Hyalomma marginatum*, *Hyalomma rufipes*, *H. impeltatum*, *H. aegyptium*, and *Hyalomma excavatum* infested birds, Common eland, gerbils, and hedgehogs, but Scimitar-horned oryx was infested with *H. dromedarii* and *Hyalomma excavatum*. Several species of *Rhipicephalus* were associated in bush pig, Bushveld gerbil, gemsbok, leopard, and Roan antelope in the present study (Figure 2 and Supplementary Figures S1 and S2).

## 4. Discussion

Ticks are considered important vectors of pathogens responsible for diseases in both animals and humans, posing a significant threat to global public health. Tick-borne protozoan, bacterial, and viral pathogens are increasingly recognized as important causes of morbidity and mortality in animals and humans, underscoring the urgency to contribute to the elucidation of the epidemiology of tick-borne diseases. Nowadays, globalization and global warming facilitate the introduction of vectors and pathogens into non-endemic areas [9,67]. Tick-borne infectious diseases have recently been documented in ticks collected from wild animals, thanks to advances in specific molecular tools and sequencing of parasitic, bacterial, and viral pathogens [68]. Ticks are recognized as reservoirs and play important roles in the transmission of pathogens related to almost all vertebrates including humans [69]. Ticks of the genera *Amblyomma*, *Rhipicephalus*, *Hyalomma*, *Ixodes*, *Ornithodoros*, *Haemaphysalis*, and *Dermatocentor* were reported as vectors of several pathogens [70]. In this study, ticks collected from a wide variety of wild hosts revealed the presence of several pathogens including parasites, bacteria, and viruses over a wide area in Africa. Among the pathogens, *Babesia* species were found in ticks and wild animals, highlighting their potential role in the epidemiology of piroplasmosis, with possible implications for local outbreaks [71]. *Babesia bigemina* and *Babesia bovis* are the main causative agents of bovine babesiosis. *Babesia bigemina* was reported *in I. ricinus*, *Dermacentor marginatus*, *Haemaphysalis punctata*, and *Rhipicephalus bursa*, collected from common fallow deer, mouflon, and red deer [72,73]. The clinical manifestation of *Babesia bigemina* has been reported in cattle but its pathogenicity is less than that of *Babesia bovis* [74]. *Babesia bigemina* and *Babesia bovis* are associated with diseases in wild animals [75]. Some species such as *B. venatorum* that can infect humans and cause disease have been identified in mouflon [76]. Furthermore, ticks positive for *B. caballi* were found in the vegetation and wild animals [77,78] and serum samples from wild rabbits were positive for *B. caballi* [79]. As for *Babesia occultans*, the pathogen was found in ticks collected from wild boars and hares [80], but also reported in the blood of buffalo [81]. This information suggests further research is needed to demonstrate the importance of wild animals as a source of Babesia infection for ticks. In the current study, we found frequent reports of *Anaplasma* in ticks collected from wild animals and vegetation. It was identified in many tick species of the genera *Rhipicephalus*, *Amblyomma*, *Dermacentor*, *Ixodes*, and *Hyalomma* which have the potential to play a crucial role for circulation of this pathogen in nature [82]. *A. phagocytophilum* was detected in R. pulchellus collected from vegetation, and *Anaplasma platys* was found in Rhipicephalus evertsi evertsi collected from wildlife [39,65]. Previous reports have shown the presence of *A. phagocytophilum* in wild animals and they are identified as a reservoir hosts [83,84]. In addition, the species of *A. bovis*, *Anaplasma marginale*, and *Anaplasma ovis* were shown in ticks collected from roe deer and vegetation [85,86]. They are considered to be important pathogens due to the implications for animal health [87]. Ticks may play an important role in distribution and maintenance of pathogens, serving as suitable reservoirs for hosts. *Bartonella* spp. affect diverse hosts but are mainly associated with mammalian species, including domestic and wild animals, as well as humans, which serve as reservoir hosts for various *Bartonella* species [88,89]. *B. tamiae* was detected in *I. vespertilionis* collected from bats [48] but it was also isolated in the blood of patients [90]. No scientific evidence has shown the capacity of ticks to transmit *B. tamiae* to animals and humans [91]. Ghosh et al. [92] showed that vectors and reservoir hosts present a high risk of infection to livestock and humans. Species of *Borrelia* are tick-transmitted bacteria affecting a wide range of wild and domestic animals and are the causative agents of borreliosis [29,93]. It has been reported in several tick species parasitizing avian and mammalian hosts [94]. According to Sala and De Faveri [95], the ticks and wild animals play an important role in the epidemiology of borrelia diseases. As for *Coxiella burnetii*, it was identified in tick species collected from wild and domestic animals [47], but the precise vectorial role of ticks needs further elucidation. However, the presence of *C. burnetii* in ticks collected from wild animals may indicate the role that ticks and wildlife could play in the epidemiology of Q fever. In addition to *Coxiella* spp., *Ehrlichia* spp. were shown in several tick species collected from hedgehogs [96,97,98]. *E. ruminantium* was detected in *A. evertsi evertsi*, *A. gemma*, *A. sparsum*, and *A. variegatum* collected from wildlife [98]. However, *E. ruminantium* infection was detected in wild ruminants [99] and in blood of wild ungulates [100]. *E. canis* was detected in *D. marginatus* and *Ixodes canisuga* collected from shepherd dogs and red foxes, respectively [101]. The prevalence of *E. ewingii* infection was confirmed in white-tailed deer [102]. Molecular evidence of *Ehrlichia canis*, *Ehrlichia ewingii*, and *Ehrlichia muris* in humans has been reported in several countries [96,103], indicating the zoonotic potential of these agents. *Ehrlichia* species are important due to their zoonotic potential and widespread geographical distribution [96]. But also, non-zoonotic *E. ruminantium* as the cause of heartwater disease and Theileria parva as the cause of East Coast Fever can cause significant epizootics in domestic animals leading to devastating economic losses and even the shift away from cattle farming. Thus, species’ tick-borne pathogens should be given greater attention in order to protect livestock. *R. africae*, *Rickettsia massiliae*, *Rickettsia raoultii*, and *Rickettsia slovaca* were reported in ticks collected from wild animals [104]. The risk of human and domestic animal infection could be more widespread due to the ubiquitous presence of tick vectors and reservoir hosts in the wild. Generally speaking, pathogens were identified in both ticks and hosts. Risk factors influencing the prevalence of tick-borne pathogens may include the distribution of tick vectors, the abundance of animals, and their migratory movements. Tick-borne viruses were detected in *A. gemma* and *R. pulchellus* ticks collected from giraffe and warthog, thus supporting the role of wild animals in maintaining viral infections within tick populations [62]. Serologic evidence of Bunyamwera group arbovirus infections was detected in deer [62]. Tick-borne viruses are emerging pathogens described in ticks and are a topic of considerable interest in public health, including their role in humans’ fatalities [105,106,107]. It has been reported that Bunyamwera virus, Ndumu virus, Semliki Forest virus, Thogoto virus, and West Nile virus can infect mammals and humans [108,109,110]. In particular, Crimean-Congo Haemorrhagic Fever virus can cause larger outbreaks on the African continent and is a major public health concern [111,112]. Previous studies have reported African swine fever in soft ticks (*Ornithodoros*) of warthogs and the vector role they could probably play in the dynamics of the virus as the origin of the high mortality of infected pigs and wild boars [113]. Clearly, further studies are needed to fully elucidate the function of these hosts in the ecology of ticks and the viruses they harbor. Wild animals serve as suitable reservoirs for zoonotic tick-borne pathogens and hosts for ticks [28,114,115]. Studies on the ticks collected from wild animals have allowed the identification of several pathogens, thus demonstrating their involvement in the tick, host, and pathogen triangle [116,117,118]. Although few studies have detected a direct correlation between wild animals and the positive ticks, this study confirms the role of wildlife and ticks in the life cycle of pathogens [71]. Studies on pathogen infections in ticks parasitizing wild animals in Sub-Saharan Africa have been somewhat limited, despite recent reports of zoonotic infections [26,118,119]. It should be noted that the description and pathogenicity of these emerging pathogens need to be elucidated.

## 5. Conclusions

The distribution of pathogens and tick vectors is concerning, and their numbers are constantly increasing due to the rise in human activities that impact forest ecosystems, wildlife, and domestic animals. Africa is endemic to a variety of tick-borne pathogens, including viruses, bacteria, and protozoa. Most of these tick-borne diseases are zoonotic, and the host–vector–pathogen interaction is still poorly understood for most of them. Climate change, wild and domestic animals, and vectors could play an important role in increasing zoonotic transmission across the continent. This study reports relevant information on infectious pathogens in wildlife and highlights the need to understand diseases and their consequences on animal and human health. Although recent research has clearly highlighted this increased distribution, more comprehensive studies are still needed to better quantify the extent of this expansion and the prevalence of pathogens, tick vectors, and hosts in high-risk areas. Such knowledge is essential for combating ticks and tick-borne diseases.

## Figures and Tables

**Table 1:** List of the pathogens identified in tick collected from wild animals in Africa.

Pathogen	Ticks	Host Species	Country	Reference
*Babesia* spp.	*Rhipicephalus muhsamae*, *Rhipicephalus pravus*, *Hyalomma aegyptium*, *Haemaphysalis parmata*, *Ixodes aulacodi*	Grasscutters, Chimpanzee, Bush pig, Leopard, Warthog, Tortoises	Ghana, Uganda, Tanzania, Tunisia, South Africa	[38,39,40,41,42]
*Babesia * ^†^	*Rhipicephalus decoloratus*, *Rhipicephalus evertsi evertsi*, *Hyalomma rufipes*	Gemsbok, Roan antelope, Common eland	South Africa	[43]
*Theileria * ^†^	*Rhipicephalus decoloratus*, *Rhipicephalus evertsi evertsi*, *Hyalomma rufipes*	Gemsbok, Roan antelope, Common eland	South Africa	[43]
*Theileria* spp.	*Rhipicephalus evertsi evertsi*, *Ixodes aulacodi*, *Amblyomma tholloni*	Grasscutters, Chimpanzee, Gemsbok, Roan antelope, Common eland, Tsessebes	South Africa, Ghana, Uganda	[38,41,44]
*Theileria separata*	*Rhipicephalus evertsi evertsi*	Tsessebes	South Africa	[44]
*Anaplasma* spp.	*Rhipicephalus sanguineus*, *Hyalomma excavatum*, *Hyalomma dromedarii*	Hedgehogs, Scimitar-horned oryx, Addax antelope	Tunisia	[45,46]
*Anaplasma* *	*Rhipicephalus decoloratus*, *Rhipicephalus evertsi evertsi* *Hyalomma rufipes*	Gemsbok, Common eland	South Africa	[43]
*Anaplasma bovis*	*Hyalomma dromedarii*	Scimitar-horned oryx	Tunisia	[46]
*Anaplasma phagocytophilum*	*Hyalomma aegyptium*, *Ixodes aulacodi*	Tortoises, Grasscutters	Tunisia, Ghana	[38,42]
*Bartonella* spp.	*Haemaphysalis erinacei*, *Rhipicephalus sanguineus*, *Amblyomma latum*, *Ixodes aulacodi*	Hedgehogs, Ball python, Snake, Grasscutters	Tunisia, Benin	[45,47]
*Bartonella tamiae*	*Ixodes vespertilionis*	Bats	Algeria	[48]
*Borrelia crocidurae*	*Ornithodoros erraticus*, *Ornithodoros* spp.	Birds, Rodent burrows	Algeria, Tunisia, Morocco	[49,50,51]
*Borrelia* spp.	*Amblyomma latum*, *Amblyomma transversale*, *Amblyomma sparsum*, *Amblyomma marmoreum*, *Amblyomma sylvaticum*, *Rhipicephalus sanguineus*, *Haemaphysalis parmata*, *Ornithodoros* spp.	Ball python, Tortoise, Hedgehogs, Birds, Chimpanzee	Ghana, Zambia, South Africa, Tunisia, Gambia, Mali, Mauritania, Morocco, Senegal, Uganda	[41,45,51,52,53]
*Borrelia lusitanae*	*Ixodes ricinus*	Monitor Lizards	Tunisia	[54]
*Candidatus* Midichloria mitochondrii	*Ixodes aulacodi*	Grasscutters	Ghana	[38]
*Candidatus* Cryptoplasma	*Amblyomma tholloni*, *Haemaphysalis parmata*	Chimpanzee	Uganda	[41]
*Coxiella* spp.	*Amblyomma marmoreum*, *Amblyomma exornatum*, *Amblyomma sylvaticum*, *Amblyomma nuttalli*	Monitor lizards, Tortoises	South Africa, Kenya	[52,55]
*Coxiella burnetii*	*Ixodes vespertilionis*, *Haemaphysalis erinacei*, *Rhipicephalus sanguineus*, *Hyalomma aegyptium*, *Ixodes* spp.	Wild boar, Hedgehogs	Algeria, Tunisia	[45,48]
*Ehrlichia* spp.	*Haemaphysalis erinacei*, *Hyalomma excavatum*, *Hyalomma dromedarii*, *Haemaphysalis* spp.	Hedgehogs, Scimitar-horned oryx, Scimitar-horned oryx, Addax antelope, Chimpanzee	Tunisia, Uganda	[41,45,46]
*Ehrlichia* *	*Rhipicephalus decoloratus*, *Rhipicephalus evertsi evertsi*, *Hyalomma rufipes*	Gemsbok, Common eland	South Africa	[43]
*Ehrlichia canis*	*Amblyomma latum*	Monitor lizards	Kenya	[55]
*Ehrlichia ewingii*	*Haemaphysalis erinacei*	Hedgehogs	Tunisia	[45]
*Ehrlichia muris*	*Ixodes aulacodi*	Grasscutters	Ghana	[38]
*Ehrlichia ruminantium*	*Amblyomma sparsum*, *Amblyomma eburneum*, *Amblyomma variegatum*, *Amblyomma falsomarmoreum*, *Amblyomma nuttalli*	Tortoises, African buffaloes	Mozambique Kenya	[55,56]
*Francisella* spp.	*Amblyomma latum*	Snake	South Africa	[52]
*Hepatozoon* spp.	*Amblyomma marmoreum*, *Ixodes aulacodi*	Monitor lizards, Tortoises, Grasscutters	South Africa Ghana	[38,52]
*Hepatozoon fitzsimonsi*	*Amblyomma falsomarmoreum*	Tortoises	Kenya	[55]
*Rhickettsia africae*	*Amblyomma variegatum*, *Amblyomma lepidum*, *Amblyomma compressum*	Buffaloes, White Rhinoceros, Pangolin	Mozambique South Africa Liberia	[56,57,58]
*Rickettisa* spp.	*Amblyomma latum*, *Amblyomma marmoreum*, *Amblyomma sylvaticum*, *Haemaphysalis erinacei* *Ixodes* spp., *Ixodes aulacodi*, *Rhipicephalus evertsi evertsi*, *Rhipicephalus muhsamae*, *Rhipicephalus pravus*, *Rhipicephalus pulchellus*, *Rhipicephalus sanguineus*, *Rhipicephalus simus*, *Rhiplcephalus appendiculatus*	Bush pig, Buffalo, Gambian pouched rat, Grasscutters, Hedgehogs, Leopard, Snake, Tortoises, Zebra	Tanzania, South Africa, Tunisia, Benin	[40,45,47,52]
*Rickettsia aeschlimannii*	*Hyalomma aegyptium*, *Hyalomma impeltatum*	Tortoises, Gerbillus	Algeria, Mauritania	[59,60]
*Rickettsia bellii*	*Ornithodoros occidentalis*, *Ornithodoros erraticus*, *Ornithodoros normandi*	Rodent burrows	Morocco, Algeria, Tunisia	[61]
*Rickettsia felis*	*Ornithodoros occidentalis*, *Ornithodoros erraticus*, *Ornithodoros normandi*	Rodent burrows	Morocco, Algeria, Tunisia	[61]
*Rickettsia hoogstraalii*	*Ornithodoros occidentalis*, *Ornithodoros erraticus*, *Ornithodoros normandi*	Rodent burrows	Morocco Algeria Tunisia	[61]
*Rickettsia lusitaniae*	*Rhipicephalus sanguineus*	Hedgehogs	Tunisia	[45]
*Rickettsia massiliae*	*Rhipicephalus sanguineus*, *Amblyomma sylvaticum*, *Rhipicephalus simus*	Wild boar, Mangoose, Hedgehog, Tortoises, Bushveld gerbil, Rüppell’s fox	Algeria, South Africa, Tunisia, Morocco	[45,48,57,60]
*Rickettsia nicoyana*	*Ornithodoros occidentalis*, *Ornithodoros erraticus*, *Ornithodoros normandi*	Rodent burrows	Morocco, Algeria, Tunisia	[61]
*Rickettsia parkeri*	*Rhipicephalus sanguineus*	African wildcat	Morocco	[60]
*Rickettsia raoultii*	*Haemaphysalis paraleachi*, *Ixodes muniensis*	Duiker	Liberia	[58]
*Rickettsia slovaca*	*Haemaphysalis punctata*, *Dermacentor marginatus*	Wild boar	Algeria	[48]
*Rickettsia wissemanii*	*Ornithodoros occidentalis*, *Ornithodoros erraticus*, *Ornithodoros normandi*	Rodent burrows	Morocco, Algeria, Tunisia	[61]
*Rickettsia asemboensis*	*Ornithodoros occidentalis*, *Ornithodoros erraticus*, *Ornithodoros normandi*	Rodent burrows	Morocco, Algeria, Tunisia	[61]
Bunyamwera virus	*Amblyomma gemma*, *Rhipicephalus pulchellus*	Giraffe, Warthog	Kenya	[62]
Crimean-Congo Haemorrhagic Fever virus	*Hyalomma aegyptium*, *Hyalomma marginatum*	Tortoises, Birds	Algeria, Morocco	[63,64]
Ndumu virus	*Rhipicephalus pulchellus*	Warthog	Kenya	[62]
Semliki Forest virus	*Rhipicephalus pulchellus*, *Amblyomma gemma*	Warthog	Kenya	[62]
Thogoto virus	*Rhipicephalus pulchellus*	Warthog	Kenya	[62]
West Nile virus	*Rhipicephalus pulchellus*, *Amblyomma gemma*	Warthog	Kenya	[62]

* Detection of double pathogens (*Ehrlichia*/*Anaplasma*). ^†^ Detection of double pathogens (*Babesia*/*Theileria*).

**Table 2:** List of pathogens identified in ticks collected from vegetation in Africa.

Pathogen	Ticks	Vegetation	Country	Reference
*Anaplasma centrale*	*Rhipicephalus evertsi evertsi*, *Hyalomma marginatum*, *Rhipicephalus pulchellus*	Vegetation	Ethiopia	[65]
*Anaplasma marginale*	*Rhipicephalus pulchellus*	Vegetation	Ethiopia	[65]
*Anaplasma ovis*	*Rhipicephalus evertsi evertsi*, *Amblyomma lepidum*, *Amblyomma* spp., *Hyalomma marginatum*	Vegetation	Ethiopia	[65]
*Anaplasma phagocytophilum*	*Rhipicephalus pulchellus*	Vegetation	Ethiopia	[65]
*Anaplasma* spp.	*Amblyomma lepidum*	Vegetation	Ethiopia	[65]
*Babesia* spp.	*Haemaphysalis parmata*, *Ixodes muniensis*	Vegetation	Uganda	[41]
*Borrelia* spp.	*Haemaphysalis parmata*	Vegetation	Uganda	[41]
*Candidatus* Cryptoplasma	*Amblyomma tholloni*, *Haemaphysalis parmata*	Vegetation	Uganda	[41]
*Ehrlichia* spp.	*Haemaphysalis punctaleachi*	Vegetation	Uganda	[41]
*Rickettsia africae*	*Amblyomma eburneum*	Vegetation	Kenya	[66]
*Rickettsia bellii*	*Rhipicephalus maculatus*	Vegetation	Kenya	[66]
*Rickettisa* spp.	*Rhipicephalus maculatus*, *Amblyomma tholloni*, *Haemaphysalis parmata*, *Ixodes muniensis*, *Ixodes rasus*, *Rhipicephalus dux*	Vegetation	Kenya Uganda	[41,66]
*Rickettsia hulinensis*	*Rhipicephalus maculatus*	Vegetation	Kenya	[66]
*Rickettsia japonica*	*Rhipicephalus maculatus*	Vegetation	Kenya	[66]
*Teileria* spp.	*Amblyomma tholloni*	Vegetation	Uganda	[41]

## Data Availability

No new data were created or analyzed in this study. Data sharing is not applicable to this article.

## Supplementary Materials

The following supporting information can be downloaded at: https://www.mdpi.com/article/10.3390/pathogens14020116/s1, Figure S1: Tick–pathogen relationships reported in the present study; Figure S2: Wildlife–tick associations reported in the present study; Table S1: Pathogens, ticks, wild animals, vegetation.
