# Supplementary material for: Tick Diversity and Distribution of Pathogen in Ticks Collected from Wild Animals and Vegetation in Africa
Source: Pathogens. 2025 Jan 25;14(2):116. doi: 10.3390/pathogens14020116 (PMC11858156; doi:10.3390/pathogens14020116)
Supplement: Supplementary file 1 [file Figures S1 and S2.pdf › Figures S1 and S2.pdf]

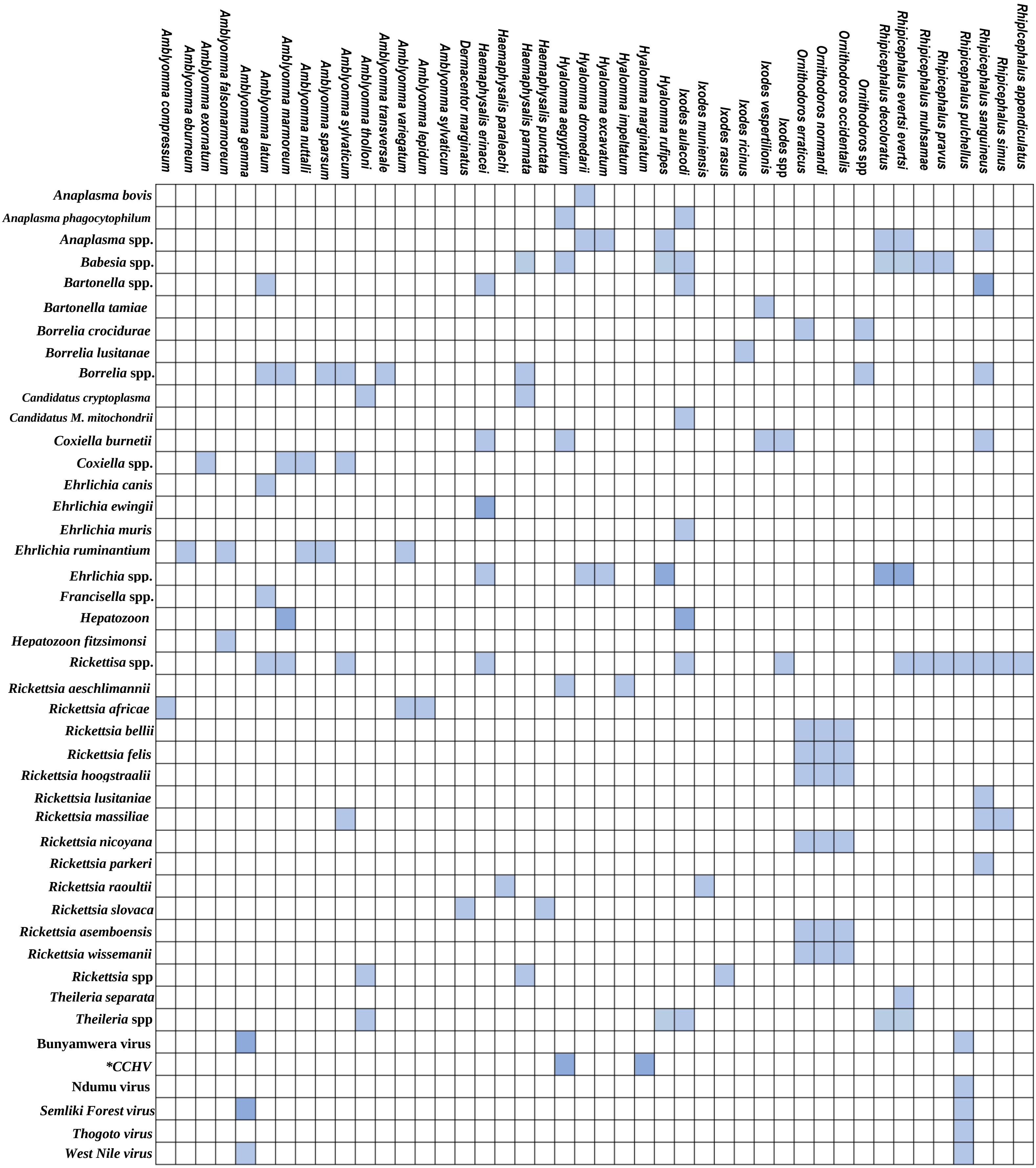

Fig. S1. Tick-pathogen relationships reported in the present study.

*Crimean-Congo Hemorrhagic Fever Virus

	Amblyomma compressum	*Amblyomma eburneum*	*Amblyomma exornatum*	*Amblyomma falsomarmoreum*	*Amblyomma gemma*	*Amblyomma latum*	*Amblyomma marmoreum*	*Amblyomma nuttalli*	*Amblyomma sparsum*	*Amblyomma sylvaticum*	*Amblyomma tholloni*	*Amblyomma transversale*	*Amblyomma variegatum*	*Amblyomma lepidum*	*Amblyomma sylvaticum*	*Dermacentor marginatus*	*Haemaphysalis erinacei*	*Haemaphysalis paraleachi*	*Haemaphysalis parmata*	*Haemaphysalis punctata*	*Hyalomma aegyptium*	*Hyalomma dromedarii*	*Hyalomma excavatum*	*Hyalomma impeltatum*	*Hyalomma marginatum*	*Hyalomma rufipes*	*Ixodes aulacodi*	*Ixodes muniensis*	*Ixodes rasus*	*Ixodes ricinus*	*Ixodes vespertilionis*	*Ixodes spp*	*Ornithodoros erraticus*	*Ornithodoros normandi*	*Ornithodoros occidentalis*	*Ornithodoros spp*	*Rhipicephalus decoloratus*	*Rhipicephalus evertsi evertsi*	*Rhipicephalus muhsamae*	*Rhipicephalus pravus*	*Rhipicephalus pulchellus*	*Rhipicephalus sanguineus*	*Rhipicephalus simus*	*Rhipicephalus appendiculatus*
**Addax antelope**																						■																						
**Ball python**						■						■																																
**Bat**																															■													
**Birds**																									■											■								
**Buffalo**		■											■																												■			
**Bush pig**																																							■				■	■
**Bushveld gerbil**																																											■	
**Chimpanze**											■								■										■															
**Common eland**																										■																		
**Duiker**																		■										■																
**African wildcat**																																										■		
**Gambian pouched rat**																																										■		
**Gemsbok**																																					■	■						
**Gerbill*us***																								■																				
**Giraffe**					■																																							
**Grasscutters**																											■															■		
**Hedgehogs**																	■				■											■										■		
**Leopard**																																								■				
**Mangoose**																																										■		
**Monitor lizards**			■			■	■																							■														
**Pangolin**	■																																											
**Rhinocéros blanc**														■																														
**Roan antelope**																																						■						
**Rodent burrows**																																	■	■	■									
**Scimitar-horned *oryx***																						■	■																					
**Snake**						■																																						
**Tortoises**				■			■	■	■	■											■																							
**Tsessebes**																																						■						
**Rüppell's fox**																																										■		
**Warthog**					■																																			■	■			
**Wild boar**																■				■											■											■		
**Zebra**																																						■			■			

Fig. S2. Wildlife-tick associations reported in the present study
